# Short‑ and long‑term outcomes after laparoscopic and open pancreatoduodenectomy for elderly patients: a propensity score‑matched study

**DOI:** 10.1186/s12877-024-05063-5

**Published:** 2024-05-27

**Authors:** Shuai Xu, Xin Deng, Shulin Wang, Guangsheng Yu, Jun Liu, Wei Gong

**Affiliations:** 1 Department of Liver Transplantation and Hepatobiliary Surgery, Shandong Provincial Hospital Affiliated to Shandong First Medical University, No.324, Jingwu Road, Jinan, Shandong 250021 China; 2 Department of Rehabilitation Medicine, The 960th Hospital of the PLA Joint Logistics Support Force, Jinan, Shandong 250031 China

**Keywords:** Laparoscopic pancreatoduodenectomy, Open pancreatoduodenectomy, Elderly patients, Pancreatic ductal adenocarcinoma, Propensity score matching

## Abstract

**Background:**

The feasibility and safety of laparoscopic pancreatoduodenectomy (LPD) in elderly patients is still controversial. This study aimed to compare the clinical outcomes of LPD and open pancreatoduodenectomy (OPD) in elderly patients.

**Methods:**

Clinical and follow-up data of elderly patients (≥ 65 years) who underwent LPD or OPD between 2015 and 2022 were retrospectively analyzed. A 1:1 propensity score-matching (PSM) analysis was performed to minimize differences between groups. Univariate and multivariate logistic regression analysis were used to select independent prognostic factors for 90-day mortality.

**Results:**

Of the 410 elderly patients, 236 underwent LPD and 174 OPD. After PSM, the LPD group had a less estimated blood loss (EBL) (100 vs. 200 mL, *P* < 0.001), lower rates of intraoperative transfusion (10.4% vs. 19.0%, *P* = 0.029), more lymph node harvest (11.0 vs. 10.0, *P* = 0.014) and shorter postoperative length of stay (LOS) (13.0 vs. 16.0 days, *P* = 0.013). There were no significant differences in serious complications, reoperation, 90-day readmission and mortality rates (all *P* > 0.05). Multivariate logistic regression analysis showed that post-pancreatectomy hemorrhage (PPH) was an independent risk factor for 90-day mortality. Elderly patients with pancreatic ductal adenocarcinoma (PDAC) who underwent LPD or OPD had similar overall survival (OS) (22.5 vs.20.4 months, *P* = 0.672) after PSM.

**Conclusions:**

It is safe and feasible for elderly patients to undergo LPD with less EBL and a shorter postoperative LOS. There was no statistically significant difference in long-term survival outcomes between elderly PDAC patients who underwent LPD or OPD.

**Supplementary Information:**

The online version contains supplementary material available at 10.1186/s12877-024-05063-5.

## Introduction

As the global population continues to age, the incidence of pancreatic and peri-ampullary tumors has increased [1–3]. Pancreatoduodenectomy (PD) is the standard surgical method for the treatment of pancreatic head and periampullary tumors [4, 5]. However, PD is a challenging abdominal operation associated with high rates of morbidity and mortality [5], and elderly patients have more comorbidities before surgery, increasing the risk of surgery [6].

Minimally invasive surgery, represented by laparoscopy, has continued to grow in recent years with the continuous improvement of minimally invasive techniques and the development of surgical equipment, and has expanded into the PD field [7, 8]. Multiple studies have shown that after the learning curves, laparoscopic pancreatoduodenectomy (LPD) has the advantages of less intraoperative estimated blood loss (EBL), shorter postoperative length of stay (LOS), and faster postoperative recovery compared to traditional open pancreatoduodenectomy (OPD) [9–11]. However, most previous studies focused on entire populations and were mostly limited to comparisons of short-term outcomes, lacking relevant studies of long-term survival outcomes. It is not yet known whether elderly patients, due to the inherent characteristics of this population, will also significantly benefit from LPD [12, 13].

The cut-off age for elderly patients varies widely in literature [14–16]. “World Population Ageing 2019 Highlights” published by the World Health Organization (WHO) states that elderly people are defined as those aged 65 years or more [17]. Additionally, studies have shown that > 60% of patients who undergo general surgery are aged > 65 years [16]. Thus, this time elderly patients were defined as those 65 or older at the time of surgery, based on previous reports [16–18].

In the present study, we retrospectively analyzed the clinical and follow-up data of elderly patients (≥ 65 years old) with benign and malignant tumors of the pancreatic head and peri-ampulla undergoing LPD or OPD in our center, and intended to compare the short-term treatment and long-term survival outcomes of these patients, in order to provide references for future clinical treatment.

## Materials and methods

### Patients and surgical procedures

This study retrospectively analyzed clinical and follow-up data on elderly patients with pancreatic head and periampullary tumors who underwent radical LPD or OPD between January 2015 and December 2022 at the Department of Liver Transplantation and Hepatobiliary Surgery of Shandong Provincial Hospital. All surgeons in the present study had passed the learning curve of LPD (defined as > 40 LPD cases according to the criteria of previous reports [19–21]). Additionally, all surgeons at our center performed lymphadenectomy in strict compliance with the domestic and international guidelines [22, 23]. Patients were divided into LPD and OPD groups based on surgical procedure. All minimally approach (except for conversion to laparotomy) were complete LPD, and the surgical procedure had been reported in detail in previous studies of our center [24, 25]. This study was approved by the Medical Ethics Committee of Shandong Provincial Hospital (No.2022 − 178), and all patients gave informed consent and signed written informed consent.

### The inclusion and exclusion criteria

The inclusion criteria were patients (1) aged ≥ 65 years; (3) underwent LPD or OPD; (2) with benign, premalignant, or resectable malignant tumors of the pancreatic and periampullary region; (4) with an American Society of Anesthesiologists (ASA) grade I-III; (5) with no history of previous major upper abdominal surgery or other malignancies. The exclusion criteria were patients: (1) with a history of other malignancies or distant metastases; (2) data missing or lost to follow-up; (3) preoperative neoadjuvant therapy. (4) death due to other non-tumor or complication causes.

### Preoperative assessment and follow-up

Preoperative examinations included complete blood count, liver function test, coagulation index, serum carbohydrate antigen 19 − 9 (CA19-9), carcinoma embryonic antigen (CEA), and carbohydrate antigen 125 (CA125). Imaging tests include chest X-rays and enhanced computed tomography (CT) or magnetic resonance imaging (MRI) of the abdomen. Patients were followed up regularly after surgery. Examination during follow-up included serum CA19-9, CEA, CA125 levels, liver function, contrast-enhanced CT or MRI every 2–3 months for the first and second years, and every 6 months thereafter until death or loss of follow-up. Tumor recurrence was diagnosed based on elevated serum tumor markers and typical CT or MRI enhanced imaging findings. After the diagnosis of the recurrence of the tumor, the patient was appropriately treated according to their general condition and the manner of the recurrence of the tumor. The 90-day mortality rate was defined as the rate of death due to tumor or post-operative complications within 90 days of surgery. Overall survival (OS) was calculated from the date of PD to either the date of death or the date of the last follow-up. The final follow-up date for the study was June 30, 2023.

### Statistical analysis

Continuous variables were expressed as medians and interquartile range (IQR) or mean ± standard deviation (SD). Variables that were normally distributed were tested by the Student’s T test, while those that did not fit the normal distribution were tested by the Mann-Whitney U test. The categorical variables were displayed as numbers and percentages. The Chi-squared test or Fisher exact probability test was used to compare the differences between the groups. The survival curves were generated using the Kaplan-Meier method and the differences between groups were compared using a log-rank test. A 1:1 propensity score matching (PSM) was performed using the nearest-neighbor matching method to minimize the differences in baseline characteristics between LPD and OPD groups [26]. A caliper radius equal to a standard deviation of 0.2 was set to prevent poor matching. All tests were two-tailed and a *P* value < 0.05 was considered statistically significant. All statistical analyses were performed with SPSS software (IBM SPSS Statistics, version 22.0; IBM Corporation, Armonk, NY, USA).

## Results

### Patients’ characteristics

**Fig. 1:**
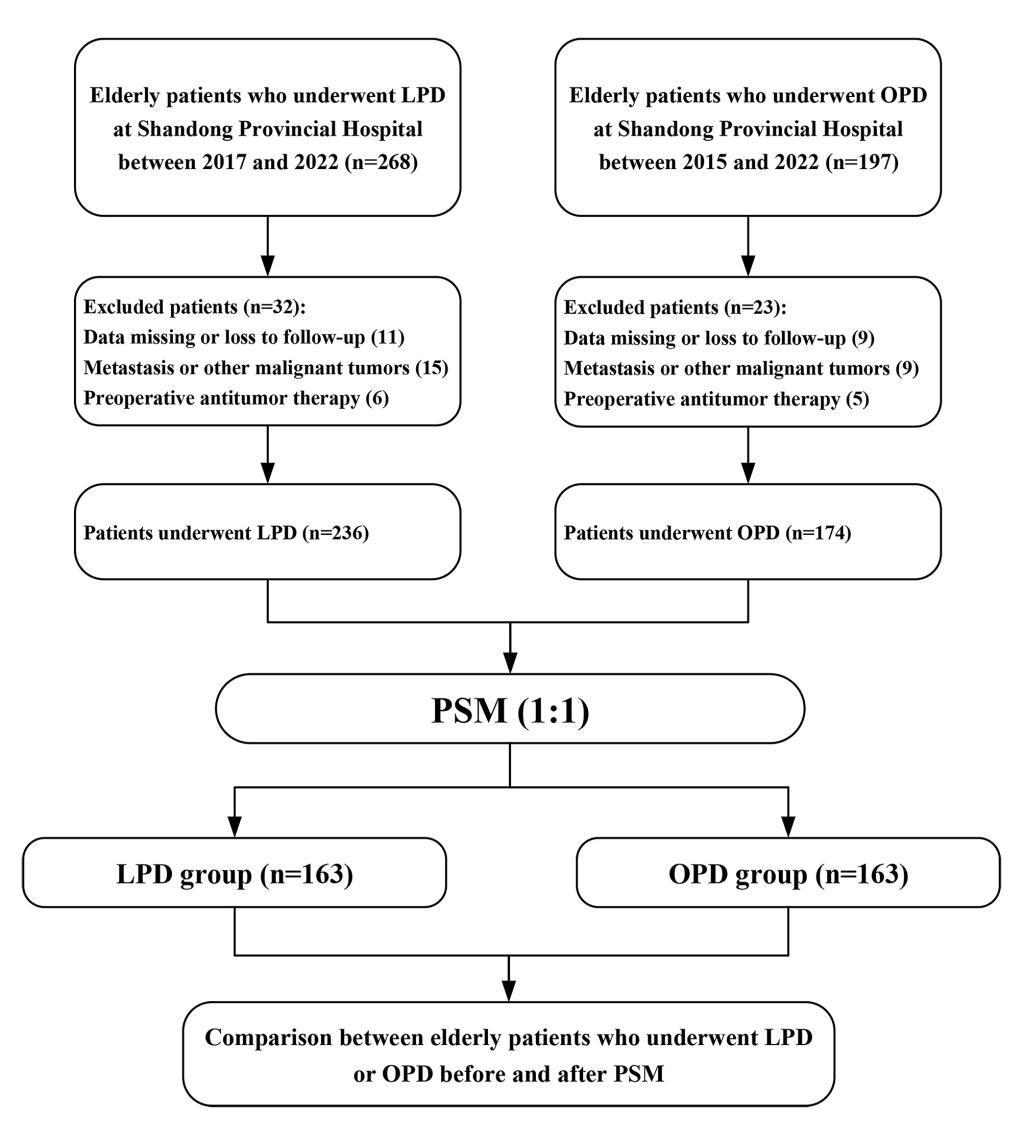
Flow chart of this study showing the selection process of patients who underwent LPD or OPD. (OPD, open pancreatoduodenectomy; LPD, laparoscopic pancreatoduodenectomy; PSM, propensity score matching)

**Fig. 2:**
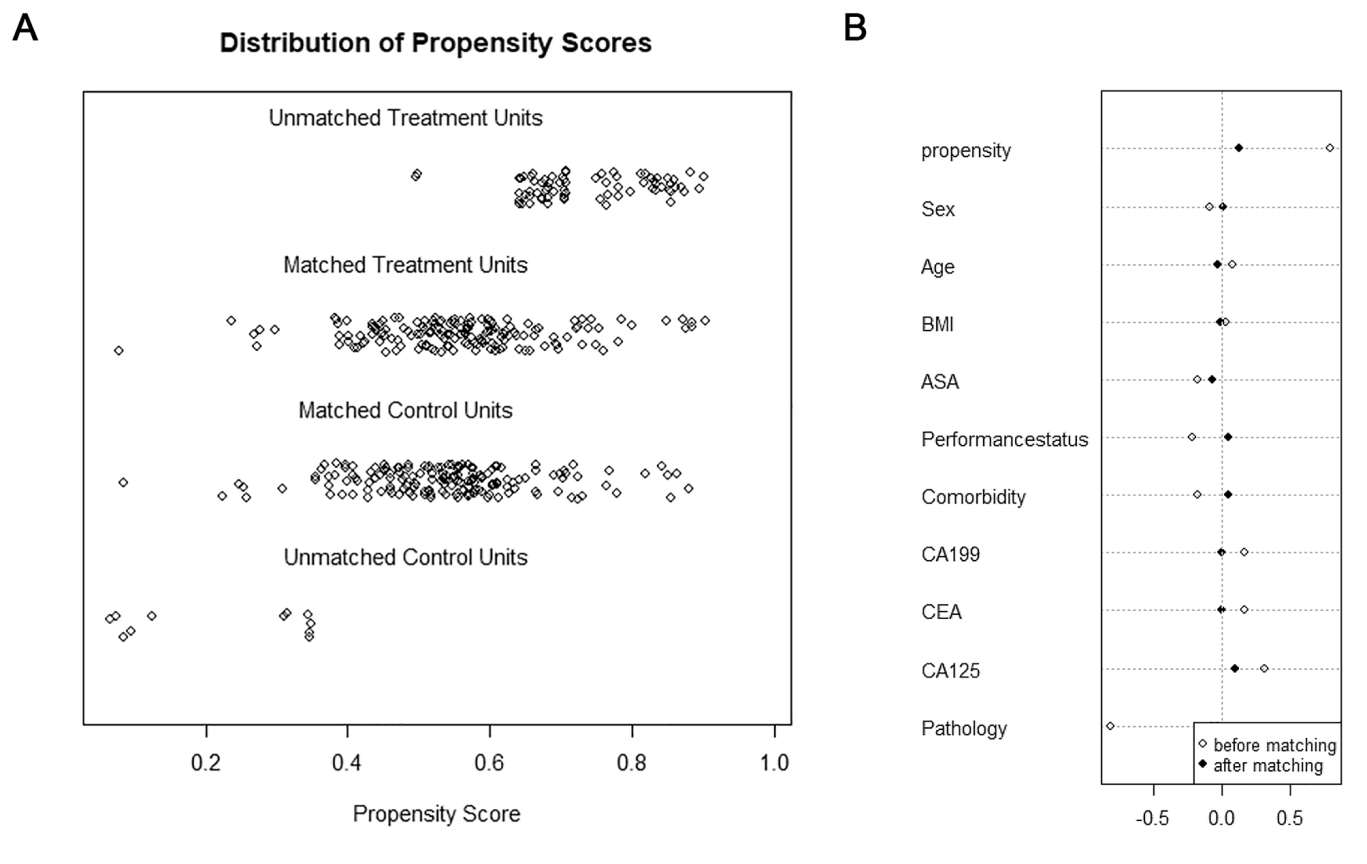
Jitter plot of individual cases and dot-plot of standardized mean differences to exhibit the results of balance test; (**A**) Jitter plot of individual cases exhibit the distribution of propensity scores before and after PSM; (**B**) dot-plot of standardized mean differences before and after PSM. (PSM, propensity score matching)

The flowchart in Fig. 1 shows the progression of LPD or OPD treatment between 2015 and 2022 for pancreatic head and periampullary tumors in the elderly patients selected for this study. There were 236 elderly patients in the LPD group and 174 in the OPD group. Before PSM, there were statistically significant differences in serum CA19-9 (*P* = 0.035), CEA (*P* < 0.001), and CA125 (*P* < 0.001) levels, and pathological diagnosis (*P* < 0.001) between the two groups. After the 1:1 PSM, each group enrolled 163 patients and the differences between groups were balanced (Table 1). Jitter plot of individual cases and dot-plot of standardized mean differences visually exhibited the results of balance test (Fig. 2A and B).

**Table 1:** Demographic and pathologic characteristics before and after propensity score matching

Variables	Before PSM (*n* = 410)		*P*-value	After PSM (*n* = 326)		*P*-value
	LPD Group (*n* = 236)	OPD Group (*n* = 174)		LPD Group (*n* = 163)	OPD Group (*n* = 163)	
Age, median (IQR), yeas	69.5 (67.0-72.8)	69.0 (67.0–73.0)	0.372	70.0 (67.0–73.0)	69.0 (67.0–73.0)	0.951
Male, N (%)	144.0 (61.0)	113.0 (64.9)	0.417	107.0 (65.6)	105.0 (64.4)	0.816
BMI, mean ± SD, kg/m^2^	23.4 ± 3.1	23.5 ± 3.6	0.740	23.4 ± 3.0	23.5 ± 3.7	0.874
ASA grade, N (%)						
≤ II	138.0 (58.5)	116.0 (66.7)	0.091	102.0 (62.6)	107.0 (65.6)	0.564
III	98.0 (41.5)	58.0 (33.3)		61.0 (37.4)	56.0 (34.4)	
Performance Status, N (%)						
0	88 (37.3)	75 (43.1)	0.067	68 (41.7)	72 (44.2)	0.536
1	86 (36.4)	70 (40.2)		59 (36.2)	63 (38.7)	
2	62 (26.3)	29 (16.7)		36 (22.1)	28 (17.2)	
Comorbidities, N (%)						
None	107 (45.3)	94 (54.0)	0.082	81 (49.7)	90 (55.2)	0.318
One or more	129 (54.7)	80 (46.0)		82 (50.3)	73 (44.8)	
CA19-9, median (IQR), U/mL	85.3 (20.7-253.7)	114.3 (38.0-408.8)	**0.035**	126.0 (33.6–297.0)	101.5 (34.9-351.5)	0.866
CEA, median (IQR), ng/mL	2.7 (1.8–4.3)	3.4 (2.4–5.5)	**< 0.001**	3.0 (2.2–4.8)	3.2 (2.4–5.2)	0.285
CA125, median (IQR), U/ml	13.8 (9.2–20.2)	17.5 (12.3–28.1)	**< 0.001**	15.0 (10.2–24.1)	17.1 (11.9–26.1)	0.081
Pathological diagnosis, N (%)						
PDAC	55 (23.3)	70 (40.2)	**< 0.001**	54 (33.1)	62 (38.0)	0.501
Cholangiocarcinoma	68 (28.8)	56 (32.2)		54 (33.1)	53 (32.5)	
Ampullary and duodenal carcinoma	67 (28.4)	40 (23.0)		38 (23.3)	40 (24.5)	
NET	1 (0.4)	1 (0.6)		0 (0.0)	1 (0.6)	
IPMN	7 (3.0)	2 (1.1)		4 (2.5)	2 (1.2)	
SPT	3 (1.3)	1 (0.6)		1 (0.6)	1 (0.6)	
Cystic neoplasm	6 (2.5)	1 (0.6)		4 (2.5)	1 (0.6)	
Others	29 (12.3)	3 (1.7)		8 (4.9)	3 (1.8)	

Bold text hinted that these variables were statistically significant

Abbreviation: IQR, interquartile range; SD, standard deviation; OPD, open pancreaticoduodenectomy; LPD, laparoscopic pancreaticoduodenectomy; BMI, body mass Index; ASA, American Society of Anesthesiologists; CA19-9, carbohydrate antigen19-9; CEA, carcinoembryonic antigen; CA125, carbohydrate antigen125; PDAC, pancreatic ductal adenocarcinoma; NET, neuroendocrine tumor; IPMN, intraductal papillary mucous neoplasm; SPT, solid pseudopapillary tumor

### Comparison of short-term outcomes between LPD group and OPD group before and after PSM

The perioperative results for both groups are shown in Table 2. After PSM, there were no significant differences between the LPD group and the OPD group in operative duration (305.0 vs. 295.0 min, *P* = 0.074), vascular reconstruction rate (5.5% vs. 8.0%, *P* = 0.377), R0 resection rate (93.3% vs. 95.1%, *P* = 0.478), clinically relevant-postoperative pancreatic fistula (CR-POPF) (12.3% vs. 11.7%, *P* = 0.889), delayed gastric emptying (DGE) (12.9% vs. 12.9%, *P* = 0.792), bile leakage (11.0% vs. 9.2%, *P* = 0.582), post-pancreatectomy hemorrhage (PPH) (8.0% vs. 6.7%, *P* = 0.671), serious complications (C-D grade ≥ III) (17.8% vs. 16.6%, *P* = 0.769), reoperation rate (5.5% vs. 4.3%, *P* = 0.608), 90-day readmission rate (3.7% vs. 4.3%, *P* = 0.777), and 90-day mortality (3.7% vs. 3.7%, *P* = 1.000). However, the LPD group had less estimated blood loss (EBL) (100.0 vs. 200.0 ml, *P* < 0.001), lower rates of intraoperative blood transfusion (10.4% vs. 19.0%, *P* = 0.029), more lymph node harvest (11.0 vs. 10.0, *P* = 0.014) and a shorter LOS (13.0 vs. 16.0 days, *P* = 0.013) compared with the OPD group. In addition, we performed a subgroup analysis of patients older than 70 years, which also revealed that the LPD group had less intraoperative EBL (100.0 vs. 200.0 ml, *P* < 0.001) and shorter LOS (14.0 vs. 16.0 days, *P* = 0.021) compared to the OPD group. Detailed results are presented in Supplementary Tables 1 and 2.

**Table 2:** Perioperative outcomes of the two groups before and after propensity score matching

Variables	Before PSM (*n* = 410)		*P*-value	After PSM (*n* = 326)		*P*-value
	LPD Group (*n* = 236)	OPD Group (*n* = 174)		LPD Group (*n* = 163)	OPD Group (*n* = 163)	
OT, median (IQR), min	305.0 (288.0-367.5)	295.0 (280.0-400.0)	0.303	305.0 (289.0-360.0)	295.0 (280.0-390.0)	0.074
EBL, median (IQR), ml	100.0 (50.0-200.0)	200.0 (100.0-300.0)	**< 0.001**	100.0 (50.0-200.0)	200.0 (100.0-300.0)	**< 0.001**
Lymph node harvest, median (IQR)	11.0 (8.0–17.0)	10.0 (8.0–16.0)	0.123	11.0 (8.0–17.0)	10.0 (8.0–16.0)	**0.014**
Blood transfusion, N (%)	29 (12.3)	33 (19.0)	0.062	17 (10.4)	31 (19.0)	**0.029**
Vascular reconstruction, N (%)	10 (4.2)	16 (9.2)	**0.042**	9 (5.5)	13 (8.0)	0.377
Conversion rate, N (%)	12 (5.1)	-	**-**	10 (6.1)	-	**-**
R0 resection, N (%)	224 (94.9)	166 (95.4)	0.821	152 (93.3)	155 (95.1)	0.478
PDAC	50 (90.9)	65 (92.9)		49 (90.7)	57 (91.9)	
Cholangiocarcinoma	64 (94.1)	54 (96.4)		51 (94.4)	52 (98.1)	
Ampullary and duodenal carcinoma	66 (98.5)	39 (97.5)		36 (94.8)	38 (95.0)	
Others	44 (95.7)	8 (100.0)		16 (94.1)	8 (100.0)	
CR-POPF, N (%)						
B	18 (7.6)	13 (7.5)	0.802	14 (8.6)	12 (7.4)	0.889
C	9 (3.8)	9 (5.2)		6 (3.7)	7 (4.3)	
DGE, N (%)						
B	19 (8.1)	17 (9.8)	0.831	14 (8.6)	16 (9.8)	0.792
C	7 (3.0)	5 (2.9)		7 (4.3)	5 (3.1)	
Bile leakage, N (%)	22 (9.3)	16 (9.2)	0.965	18 (11.0)	15 (9.2)	0.582
PPH, N (%)	15 (6.4)	14 (8.0)	0.509	13 (8.0)	11 (6.7)	0.671
Morbidity, Clavien ≥ III, N (%)	34 (14.4)	30 (17.2)	0.434	29 (17.8)	27 (16.6)	0.769
Postoperative LOS (d)	13.0 (10.0-18.9)	16.0 (12.0-20.5)	**0.001**	13.0 (11.0–20.0)	16.0 (12.0-20.6)	**0.013**
Reoperation, N (%)	10 (4.2)	7 (4.0)	0.914	9 (5.5)	7 (4.3)	0.608
90-day Readmission, N (%)	7 (3.0)	7 (4.0)	0.560	6 (3.7)	7 (4.3)	0.777
90-day mortality, N (%)	9 (3.8)	7 (4.0)	0.914	6 (3.7)	6 (3.7)	1.000

Bold text hinted that these variables were statistically significant

Abbreviation: IQR, interquartile range; OPD, open pancreaticoduodenectomy; LPD, laparoscopic pancreaticoduodenectomy; OT, operative time; EBL, estimated blood loss; PDAC, pancreatic ductal adenocarcinoma; CR-POPF, clinically relevant-postoperative pancreatic fistula; DGE, delayed gastric emptying; PPH, post-pancreatectomy hemorrhage; LOS, length of stay

### Perioperative outcomes in elderly patients with pancreatic ductal adenocarcinoma treated with LPD or OPD before and after PSM

Subgroup analysis demonstrated that a total of 125 elderly patients with pancreatic ductal adenocarcinoma (PDAC) underwent LPD or OPD, among which 55 underwent LPD and 70 underwent OPD. Before PSM, patients in the OPD group had larger tumor diameters (3.5 vs. 3.0 cm, *P* = 0.018), with no statistically significant differences in other baseline characteristics (Supplementary Table 3). After PSM, 46 patients were included in each group, and there were no statistically significant differences in perioperative outcomes between the LPD and OPD groups in terms of operative duration, EBL, lymph node harvest, R0 resection rate, incidence of postoperative serious complications, and 90-day mortality rate. The detailed results are presented in Supplementary Table 4.

### Long-term outcomes in elderly patients with PDAC before and after PSM

**Fig. 3:**
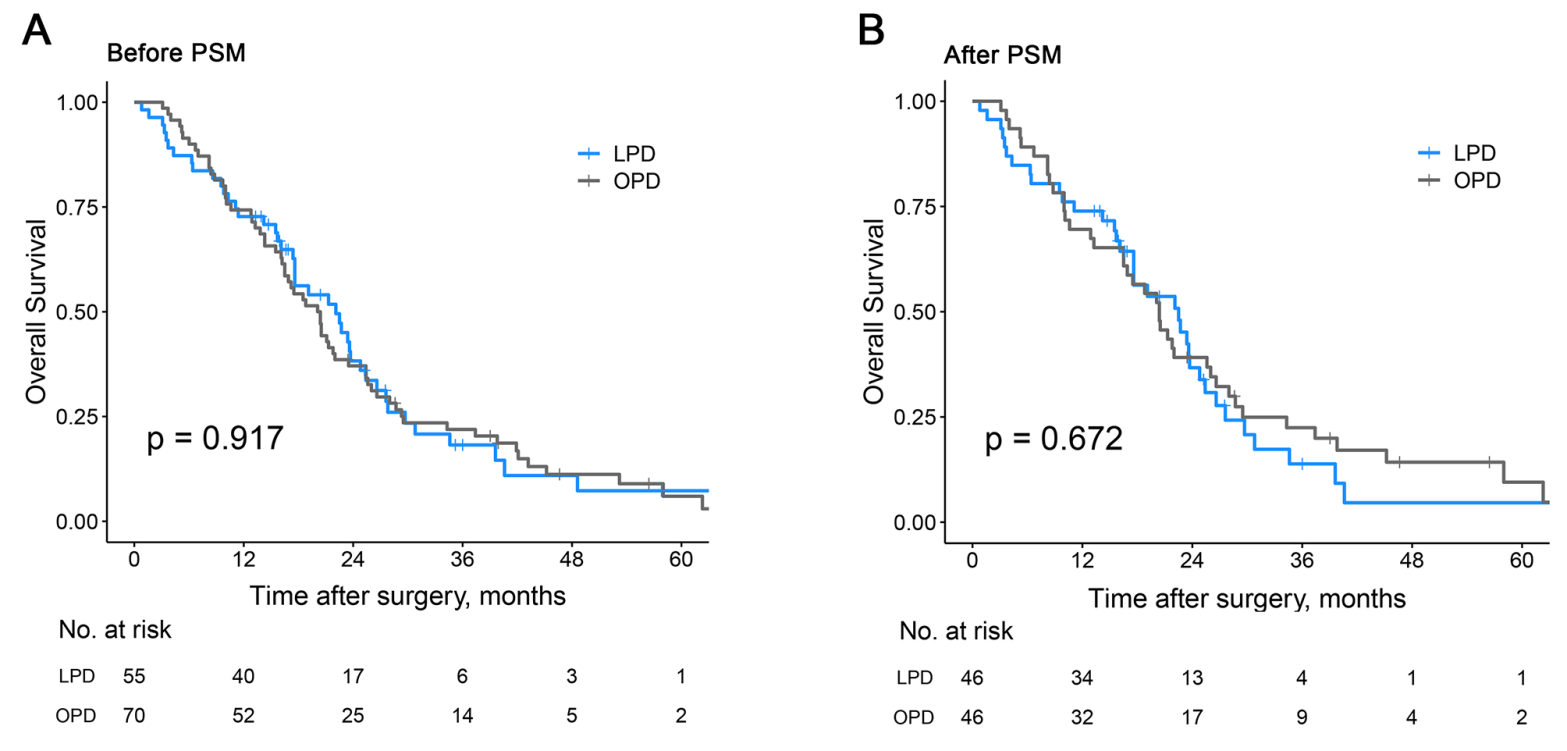
Kaplan-Meier curves estimating OS in PDAC patients who underwent LPD or OPD before and after PSM. (**A**) OS of PDAC patients before PSM; (**B**) OS of PDAC patients after PSM. (OS, overall survival; OPD, open pancreatoduodenectomy; LPD, laparoscopic pancreatoduodenectomy; PSM, propensity score matching)

Before PSM, the 1-, 3-, and 5-year survival rates of patients in the LPD group were 72.7%, 18.2%, and 7.3%, respectively, with a median OS of 22.1 (16.1–28.1) months. Patients in the OPD group had 1-, 3-, and 5-year survival rates of 74.3%, 21.9%, and 6.0%, respectively; and the median OS was 20.1 (16.7–23.5) months. There was no statistically significant difference in long-term survival outcomes between the two groups (*P* = 0.917) (Supplementary Table 5, Fig. 3A). After PSM, the 1-, 3-, and 5-year survival rates of patients in the LPD group were 73.9%, 13.9%, and 4.6%, respectively, with a median OS of 22.5 (16.1–28.9) months; Patients in the OPD group had 1-, 3-, and 5-year survival rates of 69.6%, 22.4%, and 9.5%, respectively; and the median OS was 20.4 (16.2–24.6) months. Similarly, there was no statistically significant difference in long-term survival outcomes between the two groups (*P* = 0.672) (Supplementary Table 5, Fig. 3B).

### Univariate and multivariate logistic regression analysis of 90-day mortality

Univariate logistic regression analysis showed that CR-POPF (C vs. no and BL, OR 32.182; 95% CI 9.622-107.633; *P* < 0.001), DGE (B vs. no and A, OR 6.326; 95% CI 1.997–20.044; *P* = 0.002; C vs. no and A, OR 7.844; 95% CI 1.497–41.095; *P* = 0.015), PPH (OR 24.043; 95%CI 8.121–71.177; *P* < 0.001), and reoperation (OR 14.470; 95%CI 4.344–48.195; *P* < 0.001) were the risk factors for 90-day mortality in elderly patients. And multivariate analysis showed that only PPH (OR 7.206; 95% CI 1.032–50.331; *P* = 0.046) was the independent prognostic factor for 90-day mortality in elderly patients (Table 3).

**Table 3:** Univariate and multivariate logistic regression analyses for predicting 90-day mortality of all elderly patients who underwent PD (*n* = 410)

Characteristics	Univariate analysis			Multivariate analysis		
	B	OR (95% CI)	*P*-value	B	OR (95% CI)	*P*-value
Age, per year	-0.014	0.986 (0.871–1.116)	0.825			
Gender, female vs. male	-0.601	0.548 (0.174–1.731)	0.305			
BMI, per kg/m^2^	0.081	1.084 (0.933–1.259)	0.293			
ASA grade, III vs. ≤II	-0.634	0.531 (0.168–1.675)	0.280			
Operation, OPD vs. LPD	0.056	1.057 (0.386–2.896)	0.914			
Operative time, per min	0.002	1.002 (0.996–1.007)	0.568			
Estimated blood loss, per mL	0.001	1.001 (0.999–1.003)	0.216			
Intraoperative transfusion, yes vs. no	0.658	1.931 (0.602–6.193)	0.268			
Conversion, yes vs. no	-18.030	0.000 (0.000-NA)	0.999			
POPF						
B vs. no and BL	1.249	3.488 (0.693–17.561)	0.130	0.146	1.157 (0.149–8.985)	0.889
C vs. no and BL	3.471	32.182 (9.622-107.633)	**< 0.001**	1.656	5.237 (0.689–39.794)	0.110
DGE,						
B vs. no and A	1.845	6.326 (1.997–20.044)	**0.002**	1.087	2.966 (0.622–14.130)	0.172
C vs. no and A	2.060	7.844 (1.497–41.095)	**0.015**	0.458	1.581 (0.166–15.045)	0.690
Bile leakage, yes vs. no	0.862	2.367 (0.643–8.707)	0.195			
PPH, yes vs. no	3.180	24.043 (8.121–71.177)	**< 0.001**	1.975	7.206 (1.032–50.331)	**0.046**
Reoperation, yes vs. no	2.672	14.470 (4.344–48.195)	**< 0.001**	-0.464	0.629 (0.095–4.179)	0.631
Readmission, yes vs. no	1.515	4.548 (0.928–22.281)	0.062			

Bold text hinted that these variables were statistically significant in univariate or multivariate analysis

Abbreviations: PD, pancreaticoduodenectomy; OR, odds ratio. B, coefficient; CI, confidence interval; BMI, body mass index; ASA, American Society of Anesthesiologists; OPD, open pancreaticoduodenectomy; LPD, laparoscopic pancreaticoduodenectomy; POPF, postoperative pancreatic fistula; BL, biochemical leakage; DGE, delayed gastric emptying; PPH, post-pancreatectomy hemorrhage; NA, not available

## Discussion

The current study of short- and long-term outcomes in a large cohort of elderly patients (≥ 65 years) with pancreatic and periampullary tumors who underwent LPD or OPD revealed that LPD was superior to OPD in terms of short-term outcomes (e.g., amount of EBL, number of lymph node harvest, and postoperative LOS). In addition, the study also demonstrated that the long-term survival outcomes of elderly PDAC patients were similar between the LPD and OPD groups.

The probability of malignancies in elderly people is much higher than that in younger populations, increasing the need for surgery in elderly patients with malignancies [27]. Research has shown that > 60% of patients who undergo general surgery are aged > 65 years [16]. Given the fact that older patients have higher rates of cardiopulmonary disease and are less tolerant of surgical stress than younger patients, the prognosis of elderly patients with malignancies deteriorates with age [1, 28, 29]. As the population ages, the number of elderly patients with pancreatic head and periampullary tumors is also increasing. However, PD is a challenging abdominal operation associated with high rates of morbidity and mortality [5], so the decision to perform PD in elderly patients has also become exceptionally difficult [28].

Advances in surgical techniques and improvements in post-operative management have expanded the indications for PD, making the procedure significantly safer and more feasible for elderly patients [15, 27]. In recent years, rapid progress in minimally invasive pancreatic surgery has led to many centers to report on their initial experiences on LPD [9–11]. And, since robotic surgery allows for more flexible and precise manipulation of instruments and 3D visualization, a growing number of hospitals are also experimenting with robotic pancreaticoduodenectomy (RPD) [26, 30]. Our team has also participated in a multicenter, open-label, randomized controlled trial (RCT) which showed that LPD to be associated with a shorter LOS and similar rates of short-term morbidity and mortality as OPD [25]. Moreover, a large, multicenter retrospective study demonstrated that both RPD and LPD were safe and feasible with comparable outcomes [31]. Whereas, the safety and efficacy of LPD in elderly patients who often suffer from pre-existing conditions (e.g., cardiovascular diseases) and poor functional reserve remain unclear.

In the present study, the perioperative and oncological outcomes of LPD in elderly patients were compared to OPD. Perioperative outcomes after PSM were similar in both groups, but the LPD group had less EBL, more lymph node harvesting, and lower transfusion rates than the OPD group. While some recent studies demonstrated that the OT of LPD to be significantly longer than OPD [14, 25], the present study demonstrated that in experienced hands, the operative time of LPD was not significantly different from that of OPD, which is consistent with previous study [15]. We analyze that the results of some of the earlier studies comparing LPD with OPD have been disputed due to the small sample size and the negative impact of long learning curves for LPD. The advantage of shorter OT for LPD becomes evident only with increased surgical experience. Previous studies have demonstrated that minimizing blood loss during PD has been associated with better perioperative outcomes. Specifically, reductions in blood loss have been associated with decreased rates of any- and severe-complications [32–34]. While the difference of 100 ml is not huge, it also reflects the advantages of laparoscopy. The use of laparoscopic assistance broadens the surgeon’s view of the structures surrounding the intended surgical site. More precise resection results in less bleeding [35]. In addition, previous studies reported post-operative LOS of 13.5 and 17 days for LPD and OPD in older patients [9, 36]. We also demonstrated that postoperative LOS were shorter in the LPD group compared to the OPD group (13.0 vs. 16.0 days after PSM, *P* = 0.013), suggesting that minimally invasive approaches offer many unique advantages in older patients due to less invasive surgery and rapid postoperative recovery. The discharge criteria affecting LOS in our study are based on functional recovery and may differ from those used in other trials. Given that LOS is affected by country specific sociocultural factors and healthcare policies, and influenced by discharge criteria, different pathological types of disease, surgeon’s preference, and patients’ level of self-comforting, the measure is fairly subjective [25]. Evaluating the safety and effectiveness of a surgical procedure on the basis of LOS alone is not ideal. In the current study, the 90-day postoperative mortality rate in the LPD group was 3.7% [37], consistent with previous studies and strongly suggesting that LPD is safe and feasible for elderly patients. Moreover, subgroup analysis showed no significant difference in long-term survival outcomes between the LPD and OPD groups of elderly PDAC patients. Thus, LPD is worth considering in older patients with similar oncologic benefits and safety profiles [38–40].

Recent studies have shown that age per se is not a contraindication to surgery, and that selected older patients have similar perioperative outcomes after LPD as younger patients [16]. In this study, multivariate logistic analysis also showed that advanced age was not an independent risk factor for 90-day mortality, while postoperative bleeding was. According to the International Study Group of Pancreatic Surgery (ISGPS) definition, PPH was categorized into early hemorrhage (< 24 h following the operation), which is generally regarded as a failure of the surgical procedure, and late hemorrhage (> 24 h following the operation), with diverse reasons [41]. There is a positive correlation between delayed PPH and POPF, bile leakage, gastrointestinal fistula and intra-abdominal infection [42]. The intraoperative placement of an abdominal drain may indicate POPF, bile leakage, gastrointestinal fistula, or hemorrhage, depending on the nature of the drainage fluid in the postoperative period, and can be managed accordingly. Prudent intra-operative procedures, accurate hemostasis and fluent post-operative drainage are certainly key to improving PD safety in elderly patients.

There are also several limitations to this study. First, this is a retrospective study with inherent shortcomings that do not eradicate selection bias, even though PSM was performed to minimize baseline differences. Second, all the patients enrolled in this study were from China, and the clinical efficacy of laparoscopic versus open approach for elderly patients of different ethnic groups needs to be further investigated in the future. Third, as PDAC patients receiving neoadjuvant therapy prior to surgery were not included in this study, the clinical efficacy of neoadjuvant therapy in these patients was not investigated, which may have increased the bias of this study. Finally, this is a single-center report with a small sample size, and we will initiate better designed multicenter and prospective studies to compare LPD with OPD in older patients in the future.

## Conclusions

In conclusion, this study demonstrated the safety and feasibility of LPD in elderly patients with less EBL and shorter postoperative LOS. There was no statistically significant difference in long-term survival outcomes between elderly PDAC patients who underwent LPD or OPD.

### Electronic supplementary material

Below is the link to the electronic supplementary material.

Supplementary Material 1

## Data Availability

The datasets used and/or analyzed during the current study are available from the corresponding author on reasonable request.

## Abbreviations

LPD: laparoscopic pancreaticoduodenectomy
OPD: open pancreaticoduodenectomy
PD: pancreaticoduodenectomy
PSM: propensity score-matching
EBL: estimated blood loss
LOS: length of stay
PPH: post-pancreatectomy hemorrhage
PDAC: pancreatic ductal adenocarcinoma
WHO: World Health Organization
ASA: American Society of Anesthesiologists
OS: overall survival
CA19-9: carbohydrate antigen 19 − 9
CEA: carcinoma embryonic antigen
CA125: carbohydrate antigen 125
CT: computed tomography
MRI: magnetic resonance imaging
SD: standard deviation
IQR: interquartile range
CR-POPF: clinically relevant-postoperative pancreatic fistula
DGE: delayed gastric emptying
OR: odds ratio
CI: confidence interval
RCT: randomized controlled trial
ISGPS: International Study Group of Pancreatic Surgery
RPD: robotic pancreaticoduodenectomy
