# Supplementary material for: Short‑ and long‑term outcomes after laparoscopic and open pancreatoduodenectomy for elderly patients: a propensity score‑matched study
Source: BMC Geriatr. 2024 May 27;24:462. doi: 10.1186/s12877-024-05063-5 (PMC11129404; doi:10.1186/s12877-024-05063-5)
Supplement: Supplementary file 1 — Supplementary Material 1 [file 12877_2024_5063_MOESM1_ESM.docx]

[**Supplementary materials**](javascript:;)

.

**Supplementary Table 1. Demographic and pathologic characteristics of patients older than 70 years before and after propensity score matching.**

| **Variables** | **Before PSM (n=170)** | | ***P*-value** | **After PSM (n=118)** | | ***P-*value** |
| --- | --- | --- | --- | --- | --- | --- |
|  | **LPD Group (n=97)** | **OPD Group (n=73)** |  | **LPD Group (n=59)** | **OPD Group (n=59)** |  |
| Age, median (IQR), yeas | 73.0 (72.0-76.0) | 74.0 (72.0-76.0) | 0.358 | 73.0 (71.0-76.0) | 74.0 (72.0-75.0) | 0.489 |
| Male, N (%) | 60 (61.9) | 46 (63.0) | 0.877 | 38 (64.4) | 39 (66.1) | 0.847 |
| BMI, mean±SD, kg/m^2^ | 23.3±3.1 | 23.4±3.7 | 0.730 | 23.6±3.3 | 22.8±3.5 | 0.226 |
| ASA grade, N (%) |  |  |  |  |  |  |
| ≤ II | 52 (53.6) | 39 (53.4) | 0.981 | 30 (50.8) | 31 (52.5) | 0.854 |
| III | 45 (46.4) | 34 (46.6) |  | 29 (49.2) | 28 (47.5) |  |
| Performance Status, N (%) |  |  |  |  |  |  |
| 0 | 35 (36.1) | 32 (43.8) | 0.355 | 22 (37.3) | 25 (42.4) | 0.840 |
| 1 | 33 (34.0) | 26 (35.6) |  | 21 (35.6) | 20 (33.9) |  |
| 2 | 29 (29.9) | 15 (20.5) |  | 16 (27.1) | 14 (23.7) |  |
| Comorbidities, N (%) |  |  |  |  |  |  |
| None | 42 (43.3) | 37 (50.7) | 0.339 | 27 (45.8) | 29 (49.2) | 0.712 |
| One or more | 55 (56.7) | 36 (49.3) |  | 32 (54.2) | 30 (50.8) |  |
| CA19-9, median (IQR), U/mL | 105.7 (36.1-325.0) | 146.1 (47.3-409.5) | 0.315 | 131.0 (54.6-297.0) | 164.5 (55.6-433.8) | 0.343 |
| CEA, median (IQR), ng/mL | 2.9 (2.1-4.3) | 3.2 (2.2-5.8) | 0.102 | 3.0 (2.2-4.6) | 3.2 (2.1-5.2) | 0.589 |
| CA125, median (IQR), U/ml | 14.4 (10.5-24.1) | 17.6 (12.7-26.5) | **0.049** | 14.4 (10.4-23.8) | 16.1 (12.8-24.8) | 0.074 |
| Pathological diagnosis, N (%) |  |  |  |  |  |  |
| PDAC | 27 (27.8) | 30 (41.1) | **0.003** | 20 (33.9) | 22 (37.3) | 0.733 |
| Cholangiocarcinoma | 24 (24.7) | 22 (30.1) |  | 18 (30.5) | 18 (30.5) |  |
| Ampullary and duodenal adenocarcinoma | 27 (27.8) | 19 (26.0) |  | 18 (30.5) | 17 (28.8) |  |
| NET | 1 (1.0) | 0 (0.0) |  | 1 (1.7) | 0 (0.0) |  |
| IPMN | 2 (2.1) | 0 (0.0) |  | 0 (0.0) | 0 (0.0) |  |
| SPT | 0 (0.0) | 1 (1.4) |  | 0 (0.0) | 1 (1.7) |  |
| Cystic neoplasm | 3 (3.1) | 1 (1.4) |  | 0 (0.0) | 1 (1.7) |  |
| Others | 13 (13.4) | 0 (0.0) |  | 2 (3.4) | 0 (0.0) |  |

Bold text hinted that these variables were statistically significant.

Abbreviation: IQR, interquartile range; SD, standard deviation; OPD, open pancreaticoduodenectomy; LPD, laparoscopic pancreaticoduodenectomy; BMI, body mass Index; ASA, American Society of Anesthesiologists; CA19-9, carbohydrate antigen19-9; CEA, carcinoembryonic antigen; CA125, carbohydrate antigen125; PDAC, pancreatic ductal adenocarcinoma; NET, neuroendocrine tumor; IPMN, intraductal papillary mucous neoplasm; SPT, solid pseudopapillary tumor.

# Supplementary Table 2. Perioperative outcomes of patients older than 70 years before and after propensity score matching

| **Variables** | **Before PSM (n=170)** | | ***P*-value** | **After PSM (n=118)** | | ***P*-value** |
| --- | --- | --- | --- | --- | --- | --- |
|  | **LPD Group (n=97)** | **OPD Group (n=73)** |  | **LPD Group (n=59)** | **OPD Group (n=59)** |  |
| OT, median (IQR), min | 305.0 (288.0-380.0) | 295.0 (279.0-405.0) | 0.378 | 295.0 (286.0-340.0) | 295.0 (278.0-400.0) | 0.074 |
| EBL, median (IQR), ml | 100.0 (50.0-200.0) | 200.0 (100.0-300.0) | **<0.001** | 100.0 (50.0-200.0) | 200.0 (100.0-300.0) | **<0.001** |
| Lymph node harvest, median (IQR) | 11.0 (8.0-16.0) | 11.0 (8.0-16.0) | 0.423 | 11.0 (8.0-17.0) | 11.0 (8.0-16.0) | 0.412 |
| Blood transfusion, N (%) | 16 (16.5) | 13 (17.8) | 0.822 | 7 (11.9) | 10 (16.9) | 0.432 |
| Vascular reconstruction, N (%) | 4 (4.1) | 6 (8.2) | 0.261 | 2 (3.4) | 4 (6.8) | 0.675 |
| R0 resection, N (%) | 96 (99.0) | 71 (97.3) | 0.577 | 58 (98.3) | 57 (96.6) | 1.000 |
| CR-POPF, N (%) |  |  |  |  |  |  |
| B | 7 (7.2) | 4 (5.5) | 0.209 | 3 (5.1) | 4 (6.8) | 0.245 |
| C | 3 (3.1) | 7 (9.6) |  | 1 (1.7) | 5 (8.5) |  |
| DGE, N (%) |  |  |  |  |  |  |
| B | 7 (7.2) | 7 (9.6) | 0.336 | 4 (6.8) | 7 (11.9) | 0.365 |
| C | 3 (3.1) | 0 (0.0) |  | 2 (3.4) | 0 (0.0) |  |
| Bile leakage, N (%) | 12 (12.4) | 8 (11.0) | 0.777 | 10 (16.9) | 6 (10.2) | 0.282 |
| PPH, N (%) | 6 (6.2) | 10 (13.7) | 0.097 | 3 (5.1) | 8 (13.6) | 0.113 |
| Morbidity, Clavien ≥ III, N (%) | 14 (14.4) | 17 (23.3) | 0.139 | 8 (13.6) | 14 (23.7) | 0.156 |
| Postoperative LOS (d) | 14.5 (12.0-21.0) | 16.7 (12.0-20.0) | **0.006** | 14.0 (11.0-19.0) | 16.0 (12.0-20.0) | **0.021** |
| Reoperation, N (%) | 3 (3.1) | 3 (4.1) | 0.722 | 2 (3.4) | 2 (3.4) | 1.000 |
| 90-day Readmission, N (%) | 3 (3.1) | 5 (6.8) | 0.436 | 2 (3.4) | 3 (5.1) | 0.648 |
| 90-day mortality, N (%) | 2 (2.1) | 5 (6.8) | 0.244 | 1 (1.7) | 3 (5.1) | 0.611 |

Bold text hinted that these variables were statistically significant.

Abbreviation: IQR, interquartile range; OPD, open pancreaticoduodenectomy; LPD, laparoscopic pancreaticoduodenectomy; OT, operative time; EBL, estimated blood loss; CR-POPF, clinically relevant-postoperative pancreatic fistula; DGE, delayed gastric emptying; PPH, post-pancreatectomy hemorrhage; LOS, length of stay.

**Supplementary Table 3. Demographic and pathologic characteristics of elderly patients with PDAC before and after propensity score matching**

| **Variables** | **Before (n=125)** | | ***P*-value** | **After (n=92)** | | ***P-*value** |
| --- | --- | --- | --- | --- | --- | --- |
|  | **LPD Group (n=55)** | **OPD Group (n=70)** |  | **LPD Group (n=46)** | **OPD Group (n=46)** |  |
| Age, median (IQR), yeas | 70.0 (67.0-73.0) | 70.0 (67.0-73.0) | 0.585 | 70.0 (67.0-73.0) | 70.0 (67.0-73.0) | 0.784 |
| Male, N (%) | 30 (54.5) | 43 (61.4) | 0.438 | 25 (54.3) | 26 (56.5) | 0.834 |
| BMI, mean±SD, kg/m^2^ | 22.9±3.2 | 23.2±3.7 | 0.602 | 22.9±3.4 | 23.2±3.7 | 0.642 |
| ASA grade, N (%) |  |  |  |  |  |  |
| ≤ II | 34 (61.8) | 49 (70.0) | 0.336 | 26 (56.5) | 29 (63.0) | 0.524 |
| ≥ III | 21 (38.2) | 21 (30.0) |  | 20 (43.5) | 17 (37.0) |  |
| CA19-9, median (IQR), U/mL | 157.0 (53.8-662.0) | 147.9 (38.0-520.2) | 0.489 | 164.9 (69.4-661.3) | 193.5 (49.2-560.2) | 0.815 |
| CEA, median (IQR), ng/mL | 4.0 (2.7-5.7) | 4.0 (2.7-7.3) | 0.561 | 3.8 (2.6-6.0) | 3.8 (2.8-6.6) | 0.550 |
| CA125, median (IQR), U/ml | 16.2 (10.9-25.2) | 19.2 (11.9-28.6) | 0.320 | 17.0 (11.7-27.5) | 19.2 (12.1-27.8) | 0.894 |
| Differentiation, N (%) |  |  |  |  |  |  |
| Well | 5 (9.1) | 4 (5.7) | 0.577 | 2 (4.3) | 2 (4.3) | 0.975 |
| Moderate | 33 (60.0) | 39 (55.7) |  | 30 (65.2) | 29 (63.0) |  |
| Poor | 17 (30.9) | 27 (38.6) |  | 14 (30.4) | 15 (32.6) |  |
| Tumor size, cm | 3.0 (2.5-3.5) | 3.5 (2.9-4.5) | **0.018** | 3.0 (3.0-3.6) | 3.0 (2.5-4.1) | 0.878 |
| Postoperative AT, N (%) † | 32 (58.2) | 37 (52.9) | 0.552 | 27 (58.7) | 28 (60.9) | 0.832 |

Bold text hinted that these variables were statistically significant; †, including chemotherapy, radiotherapy and immunotherapy.

Abbreviation: PDAC, pancreatic ductal adenocarcinoma; PSM, propensity score matching; SD, standard deviation; IQR, interquartile range; OPD, open pancreaticoduodenectomy; LPD, laparoscopic pancreaticoduodenectomy; BMI, body mass index; ASA grade, American Society of Anesthesiologists physical status classification; Hb, hemoglobin; WBC, white blood cell; ALB, albumin; TBIL, total bilirubin; CA19-9, carbohydrate antigen19-9; CA125, carbohydrate antigen125; CEA, carcinoembryonic antigen; AT, adjuvant therapy.

# Supplementary Table 4. Perioperative outcomes of elderly patients with PDAC before and after propensity score matching

| **Variables** | **Before (n=125)** | | ***P*-value** | **After PSM (n=92)** | | ***P*-value** |
| --- | --- | --- | --- | --- | --- | --- |
|  | **LPD Group (n=55)** | **OPD Group (n=70)** |  | **LPD Group (n=46)** | **OPD Group (n=46)** |  |
| OT, median (IQR), min | 305.0 (291.0-380.0) | 308.0 (295.0-420.0) | 0.136 | 305.0 (288.0-372.5) | 344.0 (294.5-422.5) | 0.083 |
| EBL, median (IQR), ml | 150.0 (50.0-200.0) | 200.0 (100.0-300.0) | 0.157 | 150.0 (50.0-200.0) | 200.0 (87.5-300.0) | 0.467 |
| Lymph node harvest, median (IQR) | 14.0 (10.0-19.0) | 13 (10.0-18.0) | 0.242 | 14.0 (10.0-18.0) | 13 (10.0-18.0) | 0.216 |
| Blood transfusion, N (%) | 7 (12.7) | 11 (15.7) | 0.637 | 7 (15.2) | 5 (10.9) | 0.536 |
| Conversion rate, N (%) | 6 (10.9) | - | - | 6 (13.0) | - | - |
| R0 resection, N (%) | 50 (90.9) | 65 (92.9) | 0.228 | 42 (91.3) | 42 (91.3) | 1.000 |
| CR-POPF, N (%) |  |  |  |  |  |  |
| B | 3 (5.5) | 7 (10.0) | 0.327 | 1 (2.2) | 2 (4.3) | 0.513 |
| C | 1 (1.8) | 0 (0.0) |  | 1 (2.2) | 0 (0.0) |  |
| DGE, N (%) |  |  |  |  |  |  |
| B | 4 (7.3) | 5 (7.1) | 0.428 | 3 (6.5) | 2 (4.3) | 0.677 |
| C | 0 (0.0) | 3 (4.3) |  | 0 (0.0) | 2 (4.3) |  |
| Bile leakage, N (%) | 8 (14.5) | 7 (10.0) | 0.438 | 7 (15.2) | 2 (4.3) | 0.160 |
| PPH, N (%) | 1 (1.8) | 3 (4.3) | 0.630 | 1 (2.2) | 0 (0.0) | 0.315 |
| Morbidity, Clavien ≥ III, N (%) | 5 (9.1) | 7 (10.0) | 0.864 | 4 (8.7) | 2 (4.3) | 0.677 |
| Postoperative LOS (d) | 13.0 (10.4-17.5) | 14.5 (11.0-19.0) | 0.252 | 12.5 (10.0-17.4) | 14.5 (11.0-19.3) | 0.194 |
| Reoperation, N (%) | 1 (1.8) | 1 (1.4) | 0.863 | 1 (2.2) | 0 (0.0) | 0.315 |
| 90-day Readmission, N (%) | 3 (5.5) | 4 (5.7) | 0.950 | 3 (6.5) | 3 (6.5) | 1.000 |
| 90-day mortality, N (%) | 2 (3.6) | 0 (0.0) | 0.192 | 2 (4.3) | 0 (0.0) | 0.475 |

Bold text hinted that these variables were statistically significant.

Abbreviation: PDAC, pancreatic ductal adenocarcinoma; PSM, propensity score matching; IQR, interquartile range; OPD, open pancreaticoduodenectomy; LPD, laparoscopic pancreaticoduodenectomy; OT, operative time; EBL, estimated blood loss; CR-POPF, clinically relevant-postoperative pancreatic fistula; DGE, delayed gastric emptying; PPH, post-pancreatectomy hemorrhage; LOS, length of stay.

# Supplementary Table 5. Survival analyses for all elderly PDAC patients before and after PSM

| **Time after surgery** | **Before PSM** | | |  | **After PSM** | | |
| --- | --- | --- | --- | --- | --- | --- | --- |
|  | **LPD group (n=55)** | **OPD group (n=70)** | ***P*-value** |  | **LPD group (n=46)** | **OPD group (n=46)** | ***P*-value** |
| 1-year OS rate, (%) | 72.7 | 74.3 |  |  | 73.9 | 69.6 |  |
| 3-year OS rate, (%) | 18.2 | 21.9 |  |  | 13.9 | 22.4 |  |
| 5-year OS rate, (%) | 7.3 | 6.0 |  |  | 4.6 | 9.5 |  |
| Median OS (95% CI), months | 22.1 (16.1-28.1) | 20.1 (16.7-23.5) | 0.917 |  | 22.5 (16.1-28.9) | 20.4 (16.2-24.6) | 0.672 |

Abbreviation: PDAC, pancreatic ductal adenocarcinoma; OPD, open pancreaticoduodenectomy; LPD, laparoscopic pancreaticoduodenectomy; PSM, propensity score matching; OS, overall survival; CI, confidence interval.
